# Identification of m6A regulator-mediated RNA methylation modification patterns and key immune-related genes involved in atrial fibrillation

**DOI:** 10.18632/aging.204537

**Published:** 2023-02-20

**Authors:** Peng-Fei Zheng, Sen-Yu Zhou, Chang-Qing Zhong, Zhao-Fen Zheng, Zheng-Yu Liu, Hong-Wei Pan, Jian-Qiang Peng

**Affiliations:** 1 Cardiology Department, Hunan Provincial People’s Hospital, Furong, Changsha 410000, Hunan, China; 2 Clinical Research Center for Heart Failure in Hunan Province, Furong, Changsha 410000, Hunan, China; 3 Institute of Cardiovascular Epidemiology, Hunan Provincial People’s Hospital, Furong, Changsha 410000, Hunan, China; 4 The First Affiliated Hospital of Hunan Normal University (Hunan Provincial People’s Hospital), Furong, Changsha 410000, Hunan, China

**Keywords:** atrial fibrillation, HCST, m6A, immune microenvironment, NCF2

## Abstract

The role of m6A in the regulation of the immune microenvironment in atrial fibrillation (AF) remains unclear. This study systematically evaluated the RNA modification patterns mediated by differential m6A regulators in 62 AF samples, identified the pattern of immune cell infiltration in AF and identified several immune-related genes associated with AF. A total of six key differential m6A regulators between healthy subjects and AF patients were identified by the random forest classifier. Three distinct RNA modification patterns (m6A cluster-A, -B and -C) among AF samples were identified based on the expression of 6 key m6A regulators. Differential infiltrating immune cells and HALLMARKS signaling pathways between normal and AF samples as well as among samples with three distinct m6A modification patterns were identified. A total of 16 overlapping key genes were identified by weighted gene coexpression network analysis (WGCNA) combined with two machine learning methods. The expression levels of the *NCF2* and *HCST* genes were different between controls and AF patient samples as well as among samples with the distinct m6A modification patterns. RT-qPCR also proved that the expression of *NCF2* and *HCST* was significantly increased in AF patients compared with control participants. These results suggested that m6A modification plays a key role in the complexity and diversity of the immune microenvironment of AF. Immunotyping of patients with AF will help to develop more accurate immunotherapy strategies for those with a significant immune response. The *NCF2* and *HCST* genes may be novel biomarkers for the accurate diagnosis and immunotherapy of AF.

## INTRODUCTION

Atrial fibr1illation (AF) is one of the most common permanent arrhythmia types in the general population, and its pathological changes are characterized by electrical and structural remodeling of the left atrium [1, 2]. According to relevant reports, AF affects approximately 1%-2% of the population worldwide, its prevalence is proportional to age, and the incidence rate of AF in people over 80 years old reaches 8% [3, 4]. AF can significantly increase the risk of stroke, myocardial infarction and heart failure, which brings a heavy economic burden to the patient’s family and the whole society [5]. Therefore, it is urgent to further clarify its pathogenesis and find more effective treatments. AF is considered to be a multifactorial and complex disease that is usually associated with factors such as age, obesity, hypertension, smoking, sex, diabetes and valvular heart disease [6]. However, the pathophysiology of AF has not been fully elucidated. In a recently published study, we found that the infiltration levels of activated mast cells and regulatory T cells (Tregs) were decreased and the infiltration levels of gamma delta T cells, resting mast cells and M2 macrophages were increased in AF patients compared to those in sinus rhythm (SR) individuals [7]. These results partially elucidate the infiltration of immune cells in AF and suggest that the immune mechanism plays a key role in AF. However, more studies are needed to further fully explore the mechanism of immune infiltration in AF and may help to reveal several new immunotherapies for AF.

Traditional epigenetic modification refers to the reversible modification of proteins (histones) and DNA, which can regulate gene expression without changing the genetic sequence [8]. RNA modification has gradually attracted attention; it is considered the third layer of epigenetics and involves regulation of RNA metabolism and processing [9]. Several RNA modification forms, including N1-methyladenosine (m1A), 5-methylcytosine (m5C), and N6-methyladenosine (m6A), have been found, among which the most common modification is m6A [10]. m6A modification is a homeostatic and reversible process in eukaryotic cells that is mainly regulated by a variety of m6A regulatory factors, including demethylases (erasers), binding proteins (readers), and methyltransferases (writers) [11]. Recent studies have shown that m6A modification may play a crucial role in the regulation of the immune response. Wang et al. noticed that the *HNRNPA2B1* regulator acts as a reader, can promote m6A modification and can trigger the innate immune response by recognizing viral DNA in the context of viral infection [12]. Han et al. found that the *YTHDF1* regulator acts as a reader and is involved in antigen presentation from dendritic cells to CD8+ T cells by enhancing lysosomal cathepsin translation and promoting tumor neoantigen cross-presentation and CD8+ T-cell cross-priming, thereby promoting the immune escape of tumor cells [13]. Moreover, Li et al. found that the homeostatic differentiation of T cells may be severely impaired due to the deletion of a writer, such as *METTL3,* in T cells [14]. However, no research has focused on the role of m6A in the immune microenvironment of AF. Therefore, we investigated the effect of m6A modification on the characteristics of the immune microenvironment in AF in depth and identified key immune-related genes associated with AF. The implementation of these works will help us to deeply understand the pathogenesis of AF from a completely new perspective.

## RESULTS

### Data preprocessing

**Figure 1:**
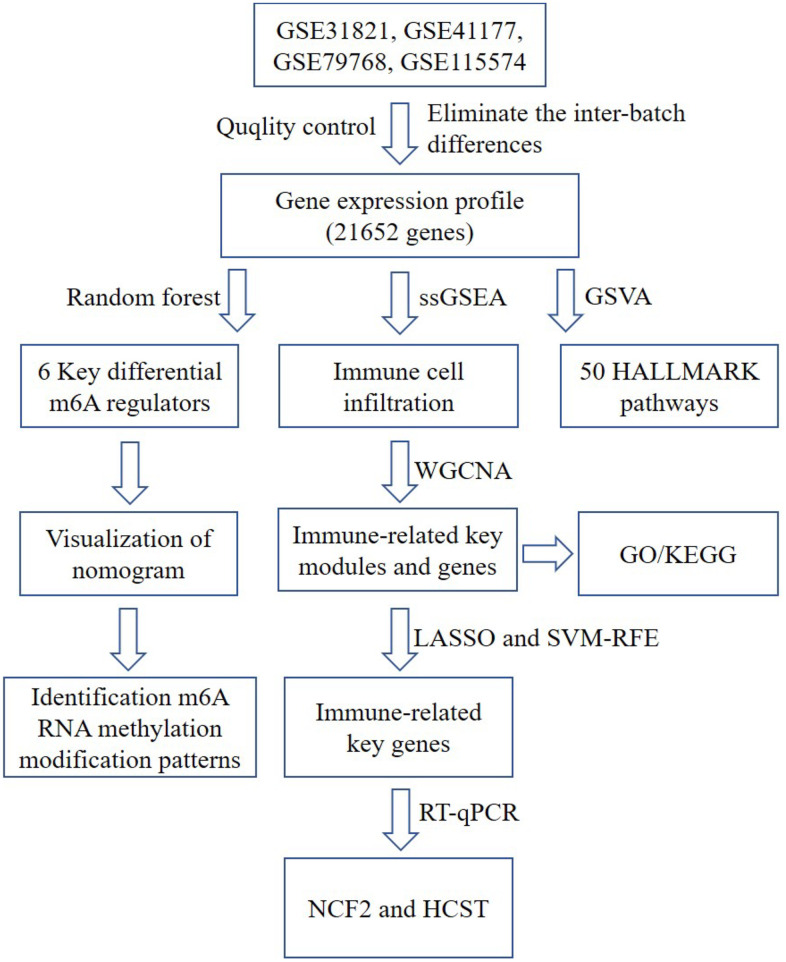
**A flow chart of the analysis.** ssGSEA, single-sample gene set enrichment analysis; GO, gene ontology annotation; KEGG, kyoto encyclopedia of genes and genomes pathway enrichment analyses; GSVA, gene set variation analysis; WGCNA, weighted gene co-expression network analysis; LASSO, Least Absolute Shrinkage and Selector Operation; SVM-RFE, Support Vector Machine-Recursive Feature Elimination; NCF2, neutrophil cytosolic factor 2; HCST: hematopoietic cell signal transducer.

The analysis process of the whole study is shown in Figure 1. The normalized gene expression matrix of the GSE31821, GSE41177, GSE79768 and GSE115574 datasets was obtained by standardizing the data format (Supplementary Figure 1A) and adding missing values by filling in the average value of the variable in all other columns. As shown in Supplementary Figure 1B, principal component analysis (PCA) of the transcriptome profile showed a significant difference between AF samples and normal samples. The integrated expression profile, including 21652 different gene symbols, was obtained from 110 atrial tissue samples after data merging and eliminating the interbatch differences between the GSE31821, GSE41177, GSE79768 and GSE115574 datasets (Supplementary Table 1).

### Identification of differential m6A regulators

**Figure 2:**
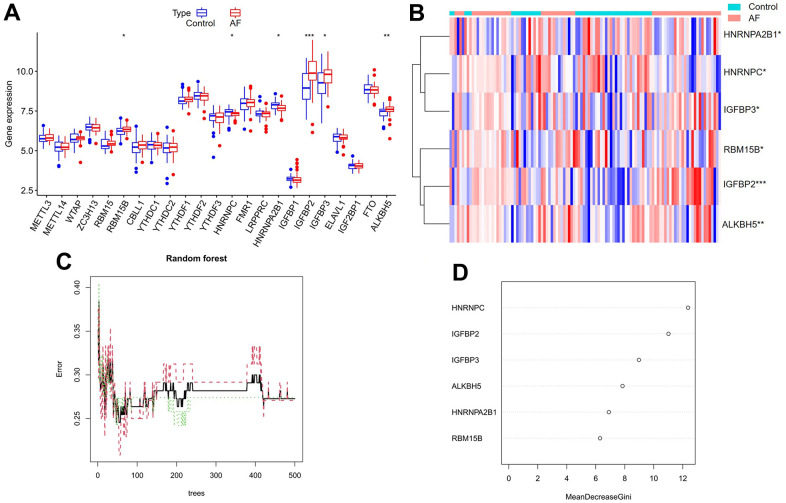
**Expression landscape of m6A RNA methylation regulators in AF and random forest model construction to identify key m6A regulators.** (**A**) Box plot of differentially expressed m6A regulators. (**B**) Heatmap of differentially expressed m6A regulators. (**C**) Plot of performance in log scale against epoch number. The x-axis represents the number of decision trees, and the y-axis indicates the error rate. When the number of decision trees is approximately 300, the error rate is relatively stable. (**D**) Results of the Gini coefficient method in the random forest classifier. The x-axis indicates the genetic variable, and the y-axis represents the importance index. *P < 0.05; **P < 0.01; ***P < 0.001.

A total of 23 different m6A regulators, including 7 writers (*METTL3*, *METTL14*, *ZC3H13*, *RBM15*, *RBM15B*, *WTAP* and *CBLL1*), 14 readers (*YTHDC1*, *LRPPRC*, *HNRNPC*, *IGFBP1*, *YTHDC2*, *HNRNPA2B1*, *IGF2BP1*, *YTHDF1*, *FMR1*, *YTHDF3*, *IGFBP2*, *YTHDF2*, *IGFBP3* and *ELAVL1*) and 2 erasers (*ALKBH5* and *FTO*), were analyzed in the current research. As shown in the box plot (Figure 2A) and heatmap plot (Figure 2B), we noticed that the expression levels of *RBM15B*, *IGFBP2*, *IGFBP3* and *ALKBH5* were significantly increased, while the expression levels of *HNRNPC* and *HNRNPA2B1* were significantly decreased in AF samples compared with SR samples.

### Random forest screening for key m6A regulators

Cyclic random forest classification was performed for all possible numbers in 1-20 variables, and the average error rate of the pattern mode was calculated. Referring to the relationship plot between the number of decision trees and the model error (Figure 2C), 300 trees were selected as the parameter of the final model, which indicates a stable error in the model. Subsequently, as shown in Figure 2D, six key m6A regulators (*RBM15B*, *IGFBP2*, *IGFBP3*, *ALKBH5*, *HNRNPC* and *HNRNPA2B1*) with importance greater than 2 were identified for subsequent analysis.

### Construction and assessment of a nomogram model

**Figure 3:**
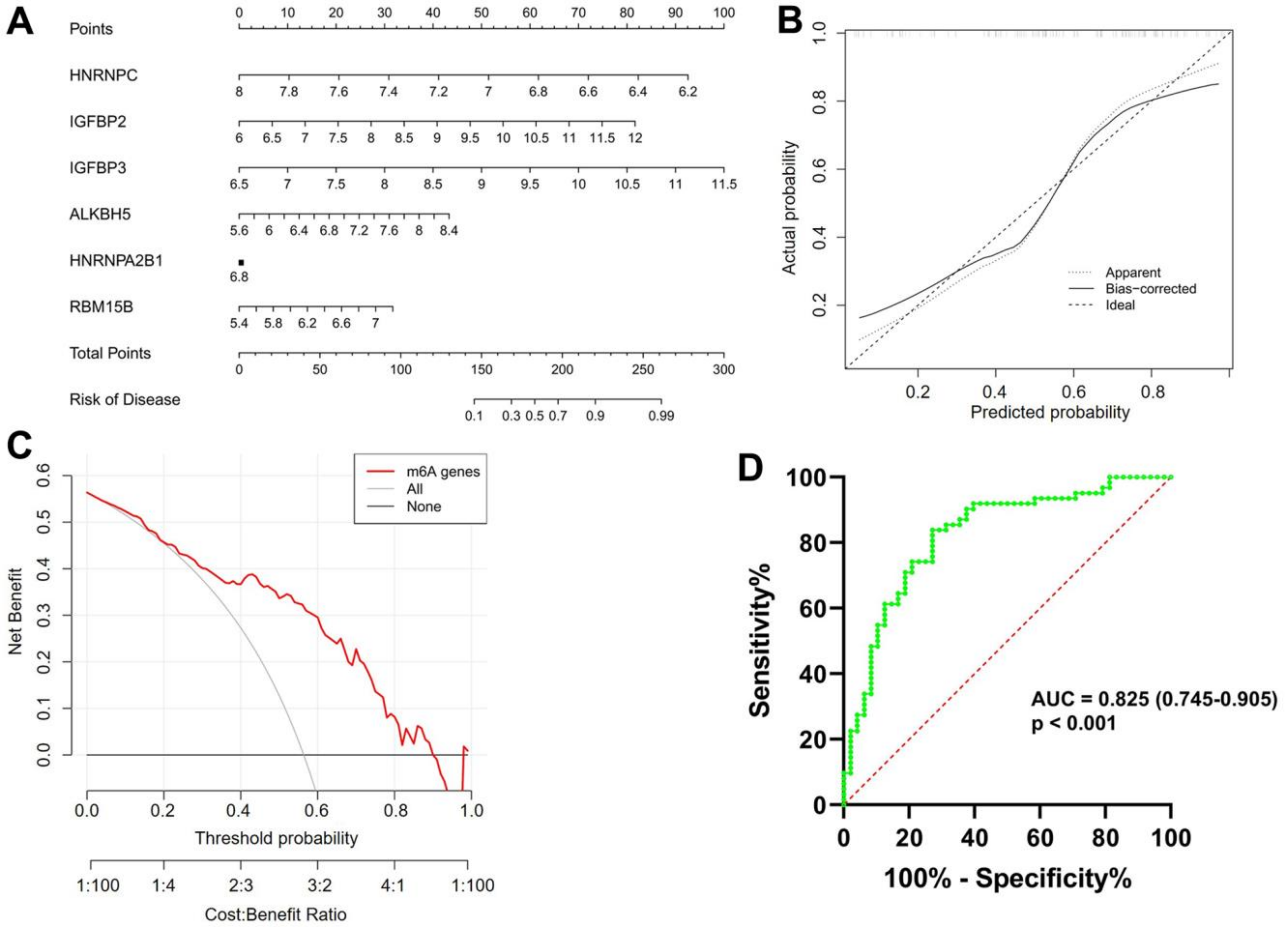
**Construction and validation of a predictive nomogram of atrial fibrillation established based on six m6A regulators.** (**A**) The nomogram of the model. (**B**) The calibration plot of the nomogram, and the diagonal dotted line represents a perfect prediction by an ideal model. (**C**) Decision curve analysis (DCA) of the nomogram. The solid line represents the performance of the nomogram, of which a closer fit to the diagonal dotted line represents a better prediction. (**D**) Receiver operating characteristic (ROC) analysis of the nomogram confirming that the model was effective in distinguishing atrial fibrillation patients from healthy subjects.

As shown in Figure 3A, a predictive nomogram was constructed based on the expression of six key m6A regulators (*RBM15B*, *IGFBP2*, *IGFBP3*, *ALKBH5*, *HNRNPC* and *HNRNPA2B1*). The calibration curve suggested that the error between the predicted and actual AF risk was very small, suggesting that the nomogram model obtained high accuracy in predicting AF (Figure 3B). Decision curve analysis (DCA) showed that the “nomogram” curve was higher than the gray line, indicating that the nomogram maintains great clinical utility in predicting the morbidity of AF patients (Figure 3C). ROC analysis reconfirmed that the model was effective in distinguishing AF patients from healthy subjects (Figure 3D).

### Identification of m6A RNA methylation modification patterns in AF

**Figure 4:**
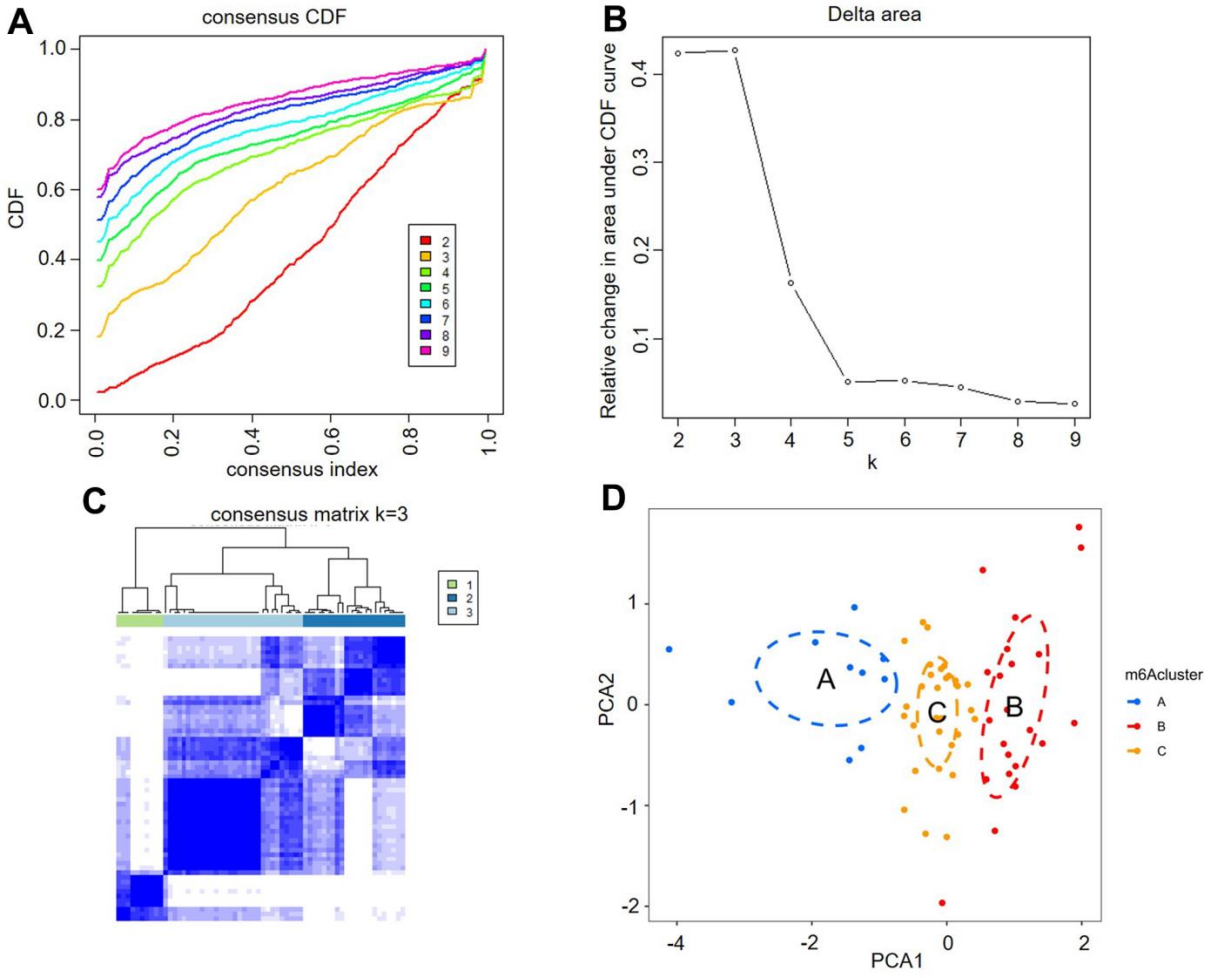
**Identification of three distinct m6A modification pattern subtypes in atrial fibrillation.** (**A**) Consensus clustering cumulative distribution function (CDF) for k = 2-9. (**B**) Relative change in the area under the CDF curve for k = 2-9. (**C**) Heatmap of the matrix of cooccurrence proportions for atrial fibrillation samples. (**D**) Principal component analysis for the transcriptome profiles of three m6A clusters, showing a remarkable difference in the transcriptome between different modification patterns.

Unsupervised consistent clustering analysis based on the expression values of six key m6A regulators in AF samples was utilized to study m6A modification patterns in AF (Figure 4A–4C). Three different subtypes of AF were identified based on qualitatively different expression of six key m6A regulators, including 10 samples in the m6A cluster-A group, 22 samples in the m6A cluster-B group and 30 samples in the m6A cluster-C group (Figure 4D and Supplementary Table 2).

### Comparison of immune microenvironment characteristics

**Figure 5:**
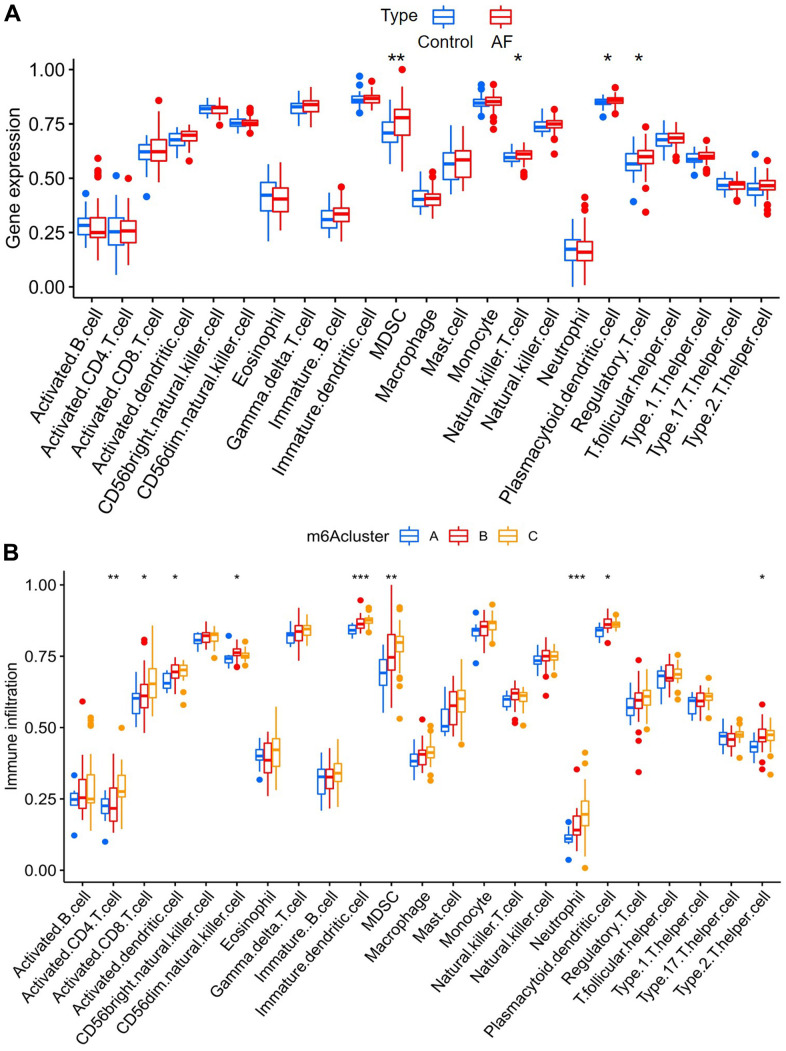
**Analysis of immune cell infiltration between different groups.** (**A**) Comparison of immunocyte abundance between controls and atrial fibrillation patients. (**B**) Comparison of immunocyte abundance in the 3 clusters.

The infiltration of many immunocytes was different between the controls and AF patients (Figure 5A), and among the AF samples with three distinct m6A modification patterns (Figure 5B). We found relatively higher infiltration of myeloid-derived suppressor cells (MDSCs), natural killer T cells, plasmacytoid dendritic cells and regulatory T cells in AF subjects than in SR patients. We also found that there was relatively higher infiltration of activated CD4 T cells, activated CD8 T cells, activated dendritic cells, CD56dim natural killer cells, immature dendritic cells, MDSCs, neutrophils, plasmacytoid dendritic cells and type-2 T helper cells in the m6A cluster-C group than in the m6A cluster-A group (Supplementary Table 3).

### Comparison of 50 HALLMARKS pathways

**Figure 6:**
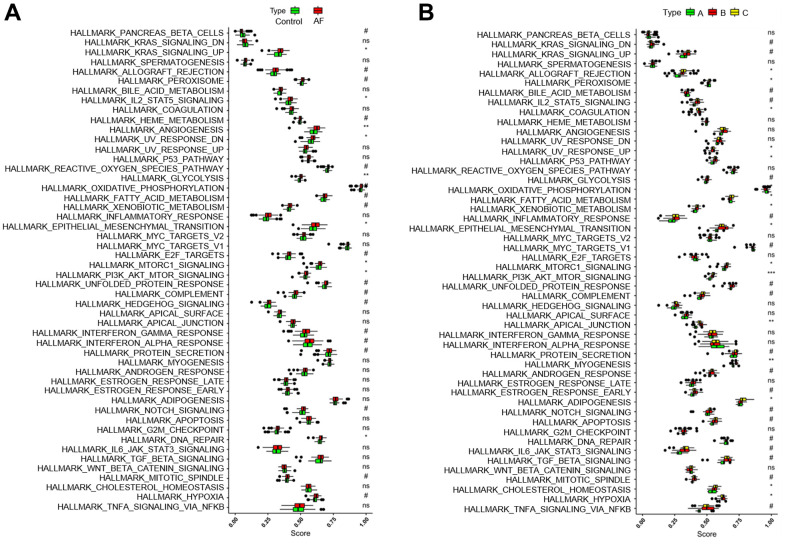
**Analysis of HALLMARKS pathway enrichment scores between different groups.** (**A**) Comparison of HALLMARKS pathway enrichment scores between controls and atrial fibrillation patients. (**B**) Comparison of HALLMARKS pathway enrichment scores in the 3 m6A clusters.

Gene set variation analysis (GSVA) was used to compare the HALLMARKS pathways between the healthy subject and AF patient samples (Figure 6A) and among the AF patient samples with three distinct m6A modification patterns (Figure 6B). Compared with the SR subjects, the AF patients had more enriched pathways, such as KRAS signaling up, IL-2/STAT5 signaling, angiogenesis, UV response down, glycolysis, epithelial mesenchymal transition, mTORC1 signaling, PI3K/Akt/mTOR signaling and DNA repair. Meanwhile, compared with the m6A cluster-A group, the m6A cluster-C group had more enriched pathways, such as allograft rejection, peroxisome, coagulation, UV response up, P53 signaling, fatty acid metabolism, xenobiotic metabolism, epithelial mesenchymal transition, mTORC1 signaling, PI3K/Akt/mTOR signaling, apical junction, myogenesis, adipogenesis, cholesterol homeostasis, and hypoxia (Supplementary Table 4).

### Identification of meaningful immune-related modules

**Figure 7:**
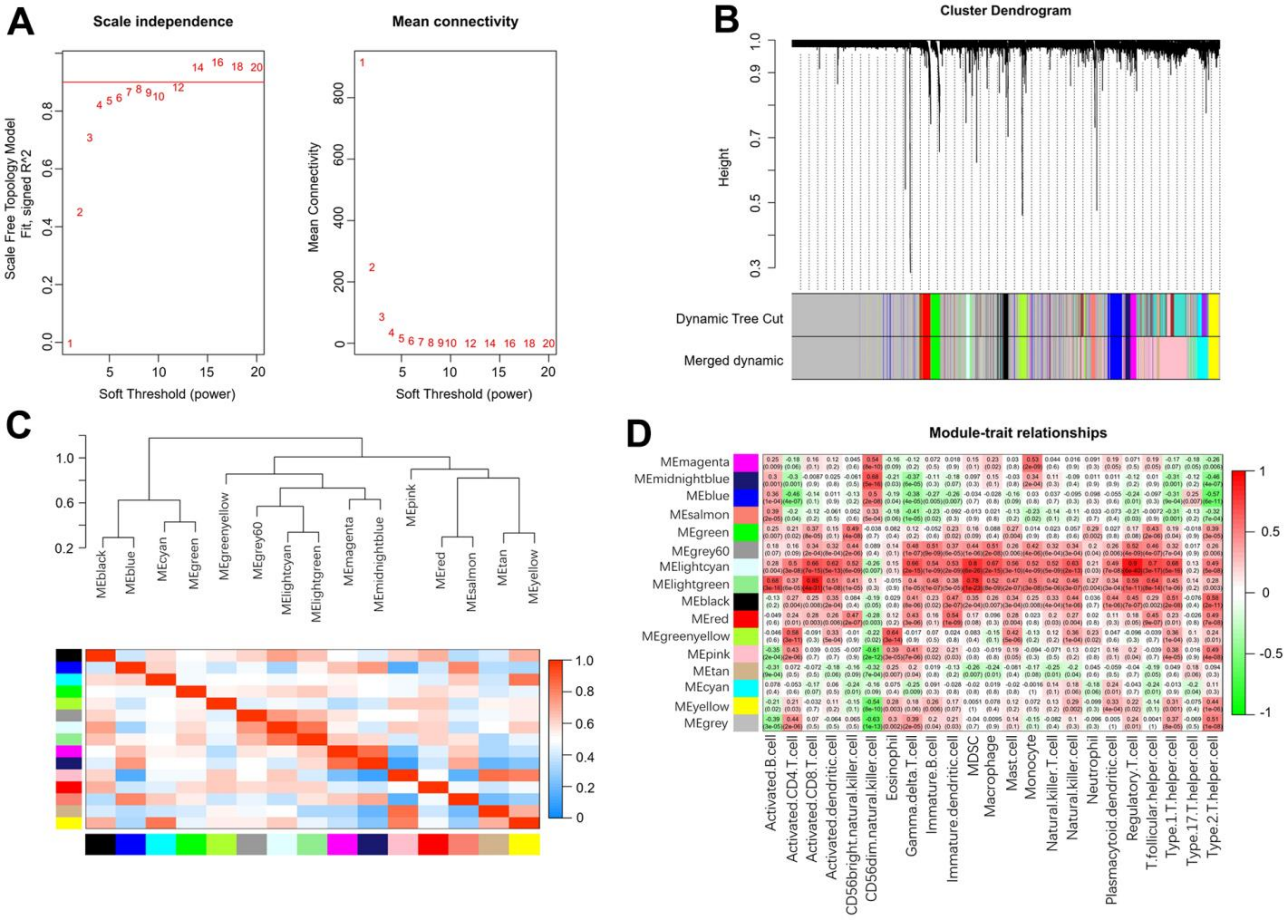
**Weighted gene coexpression network analysis.** (**A**) Analysis of network topology for various soft-thresholding powers. (**B**) Clustering dendrogram of genes. (**C**) Relationship among all the modules. (**D**) Associations between modules and infiltrating immune cells.

Weighted gene coexpression network analysis (WGCNA) was used to identify the meaningful modules that were significantly associated with infiltrating immune cells. In the WGCNA, when the correlation coefficient was greater than 0.9, the corresponding soft threshold was 14. Therefore, a soft threshold of 14 was selected to construct several gene modules (Figure 7A). Then, the topological overlap matrix combined with the hierarchical mean linkage clustering method was used to identify gene modules in each gene network. As shown in Figure 7B, a total of 15 gene modules were identified in the coexpression network. Figure 7C depicts the correlation heatmap between 15 modules. As shown in Figure 7D, the lightcyan module was highly associated with regulatory T cells (r^2^ = 0.90, *p* = 6e-40), and the lightgreen module was highly associated with activated CD8 T cells (r^2^ = 0.85, *p* = 4e-31).

### Enrichment analysis of the genes in meaningful modules

As shown in Supplementary Table 5, a total of 104 genes in the lightcyan and lightgreen modules were included in the KEGG and GO functional enrichment analyses. The top 10 biological processes, cellular components, and molecular functions are shown in Supplementary Figure 2A. The top 10 KEGG pathways are shown in Supplementary Figure 2B. A total of 104 genes were mainly enriched in the following immune-related biological processes: leukocyte-mediated immunity, positive regulation of leukocyte activation, positive regulation of cell activation, adaptive immune response based on somatic recombination of immune receptors built from immunoglobulin superfamily domains, leukocyte cell-cell adhesion, activation of immune response, leukocyte activation involved in the immune response and cell activation involved in the immune response. In addition, the details of GO and KEGG analyses are presented in Supplementary Tables 6, 7.

### Identification of key genes by machine learning

**Figure 8:**
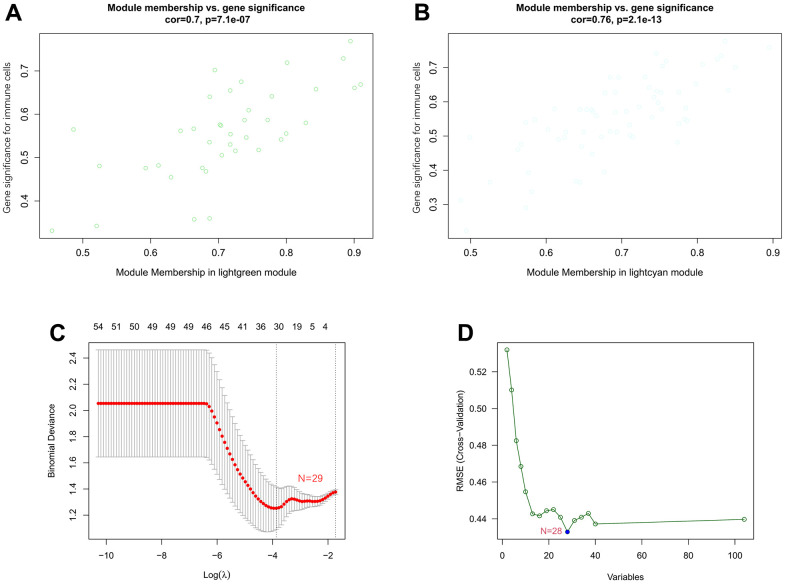
**Associations between gene significance and module membership and identification of key genes of AF by machine learning.** (**A**) Association between gene significance and module membership in the lightgreen module. (**B**) Association between gene significance and module membership in the lightcyan module. (**C**) The key genes identified by LASSO regression. (**D**) The key genes identified by the SVM-RFE algorithm.

The correlation coefficient between the color module and the gene significance was calculated to determine the significance of the module. As shown in Figure 8, we noticed that the correlation coefficients of gene significance with the lightgreen module (Figure 8A) and the lightcyan module (Figure 8B) were 0.70 (*p* = 7.1e-07) and 0.76 (*p* = 2.1e-13), respectively. Then, LASSO regression and the SVM-RFE algorithm were performed to identify several characteristic genes that were significantly associated with AF based on the expression profile of genes in the lightcyan and lightgreen modules. A total of 29 and 28 key genes were identified by LASSO regression (Figure 8C) and the SVM-RFE algorithm (Figure 8D), respectively. Moreover, a total of 16 overlapping genes were identified by both machine learning methods. The detailed gene symbols of several key genes identified by these two machine learning methods are also shown in Supplementary Table 8.

### Expression of key genes

**Figure 9:**
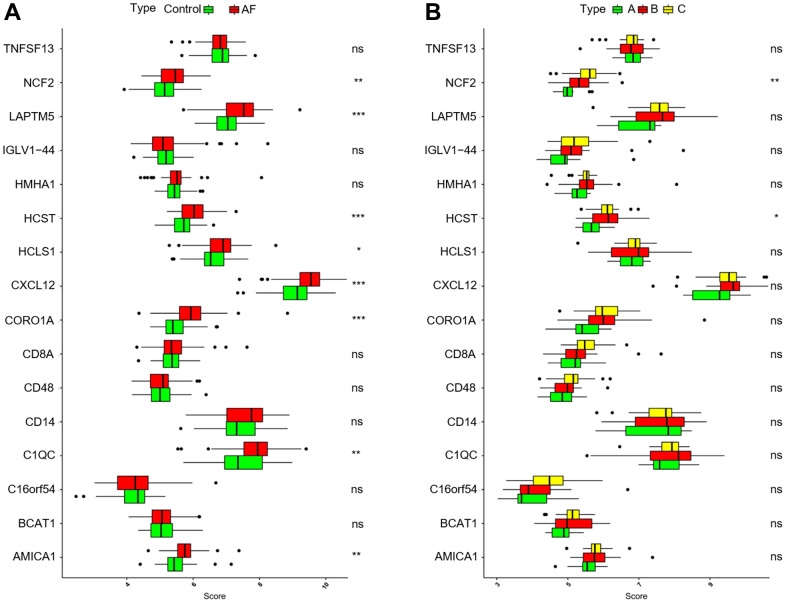
**Analysis of the differences in expression of 16 key genes in different groups.** (**A**) Comparison of the expression of 16 key genes between controls and atrial fibrillation patients. (**B**) Comparison of the expression of 16 key genes in the 3 m6A clusters. *P < 0.05; **P < 0.01; ***P < 0.001.

**Figure 10:**
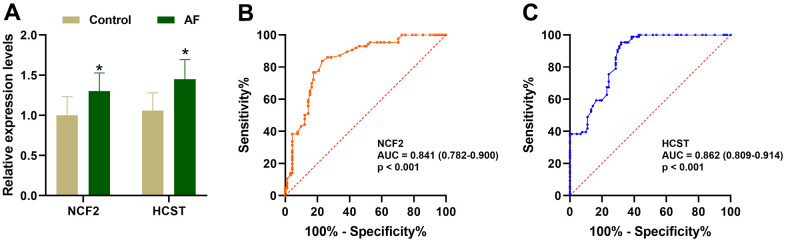
**Validation of the *NCF2* and *HCST* genes in clinical samples.** (**A**) The relative expression levels of *NCF2* and *HCST* in clinical samples. (**B**) ROC curve analysis of *NCF2*. (**C**) ROC curve analysis of *HCST*. *P < 0.05; **P < 0.01; ***P < 0.001.

As shown in Figure 9A, compared with the SR subjects, the expression levels of neutrophil cytosolic factor 2 (*NCF2*), lysosomal protein transmembrane 5 (*LAPTM5*), hematopoietic cell signal transducer (*HCST*), hematopoietic cell-specific lyn substrate 1 (*HCLS1*), C-X-C motif chemokine ligand 12 (*CXCL12*), coronin 1A (*CORO1A*), complement C1q C chain (*C1QC*) and junction adhesion molecule like (*AMICA1*) were significantly increased in AF patients. Meanwhile, compared with the m6A cluster-A group, the expression of *NCF2* and *HCST* was significantly increased in the m6A cluster-B and -C groups (Figure 9B). Since the *NCF2* and *HCST* genes differ between SR subjects and AF patients and among different m6A-modified molecular subtypes, *NCF2* and *HCST* were further verified by RT-qPCR. As shown in Figure 10A, we noticed that the expression levels of *NCF2* and *HCST* were significantly increased in AF patients compared with controls. In addition, the AUC values for *NCF2* (Figure 10B) and *HCST* (Figure 10C) were 0.841 (95% CI 0.782–0.900; *p* < 0.001) and 0.862 (95% CI 0.809–0.914; *p* < 0.001), respectively.

### Correlation between hub genes and infiltrating immune cells and 50 HALLMARKS pathways

As shown in Supplementary Figure 3A, the *NCF2* and *HCST* genes were significantly positively correlated with activated CD4 T cells, activated CD8 T cells, activated dendritic cells, CD56 bright natural killer cells, gamma delta T cells, immature B cells, immature dendritic cells, MDSCs, macrophages, mast cells, monocytes, natural killer T cells, natural killer cells, neutrophils, plasmacytoid dendritic cells, regulatory T cells, T follicular helper cells, type 1 T helper cells, and type 2 T helper cells. As shown in Supplementary Figure 3B, the *NCF2* and *HCST* genes were also significantly positively correlated with several HALLMARKS pathways, such as TNF-α signaling via NF-kB, xenobiotic metabolism, beta-catenin signaling, PI3K/Akt/mTOR signaling, the P53 pathway, mTORC1 signaling, KRAS signaling up, the interferon gamma response, the interferon alpha response, the inflammatory response, IL-2/STAT5 signaling, IL-6/JAK/STAT3 signaling, the late estrogen response, the early estrogen response, epithelial mesenchymal transition, complement, coagulation, apoptosis, apical surface, apical junction, angiogenesis, the androgen response, allograft rejection and adipogenesis.

## DISCUSSION

At present, AF is the most common persistent arrhythmia that significantly threatens the health of Chinese residents. Accumulating evidence suggests that immune and inflammatory mechanisms play a key role in the pathogenesis of AF, and these mechanisms appear to be significantly associated with the onset and persistence of AF as well as the prethrombotic state associated with AF [15, 16]. For example, Yamashita et al. demonstrated that macrophage adhesion and recruitment in the cardiac endocardium promoted inflammatory responses in human AF [17]. Hohmann et al. demonstrated that the number of inflammatory CD3+ T cells in the left atrial appendage was significantly increased in patients with SR compared to those with persistent AF [18]. On the other hand, some scholars have found that m6A modification plays an integral role in innate and adaptive immune responses [19]. Several studies have aimed to explore the role of m6A modification in immunity, especially in the infiltration of immune cells in the tumor microenvironment, and the results confirmed that m6A modification plays a fundamental role in tumor immunity [20, 21]. We also believe that the m6A modification pattern plays a similar role in the formation of the immune microenvironment in AF. However, its mechanism has not been clarified. To better clarify these issues, we systematically explored the effects of m6A modification on the infiltration of immune cells and the expression of immune-related genes in AF.

In this study, the effects of m6A modification on immune cell infiltration, inflammation, immune-related pathways, and key immune-related genes in AF were explored, and several meaningful new findings were obtained. First, we found that the expression levels of *RBM15B*, *IGFBP2*, *IGFBP3* and *ALKBH5* were significantly increased, while the expression levels of *HNRNPC* and *HNRNPA2B1* were significantly decreased in AF samples compared with SR samples, suggesting that these six key m6A regulators are involved in the development of AF. Meanwhile, a nomogram model based on the above 6 key m6A regulators suggested a diagnostic value of up to 82.5% to distinguish AF from healthy participants. This meaningful model will help us to screen high-risk individuals susceptible to atrial fibrillation at an early stage in clinical practice, and these m6A regulators may be potential novel molecular targets to help diagnose or treat AF. Second, we identified three distinct m6A modification patterns (m6A cluster-A, -B and -C) using unsupervised clustering of AF samples based on the expression values of six key m6A regulators. We found that there was relatively higher infiltration of activated CD4 T cells, activated CD8 T cells, activated dendritic cells, CD56dim natural killer cells, immature dendritic cells, MDSCs, neutrophils, plasmacytoid dendritic cells and type-2 T helper cells in the m6A cluster-C group than in the m6A cluster-A group, suggesting the essential role of m6A modification in the regulation of the immune microenvironment in AF. Presently, the classification strategy of immune subtypes is widely used in the cancer field, and identifying new immune subtypes can help to formulate better treatment plans for hepatocellular carcinoma [22]. Thus, this classification strategy of immune subtypes can also help us subtype AF samples at the immune level or molecular level and help us to implement individualized immunotherapy for patients with active immune activity.

In recent years, some key molecules related to immune cells have been discovered and are considered new biomarkers to help diagnose disease or predict prognosis [23, 24]. Li et al. found that *PPBP*, *CXCL12* and *CCL4*, as novel molecular markers for the diagnosis of valvular atrial fibrillation, were positively correlated with the infiltration of various immune cells, such as neutrophils, plasma cells and resting dendritic cells [25]. Liu et al. also noticed that *BECN1*, *ATG7*, *BCL2L1* and *MYC*, as novel molecular markers for the diagnosis of valvular atrial fibrillation, were positively correlated with the infiltration of neutrophils and negatively correlated with the infiltration of memory resting CD4 T cells and T follicular helper cells. Meanwhile, they found that the fractions of memory resting CD4 T cells and T follicular helper cells were decreased, and the fractions of resting dendritic cells, plasma cells, neutrophils, and monocytes were significantly increased in AF patients compared with SR subjects [26]. These results partially elucidated the pattern of immune cell infiltration in AF and identified several new molecular markers associated with the pathogenesis of AF. However, the identification of these key genes is based on differential expression analysis of genes. Currently, WGCNA, as a more effective bioinformatics analysis method, is expected to identify key genes ignored by differential gene analysis by constructing a scale-free gene coexpression network and identifying gene modules that are significantly related to phenotypes [27]. Meanwhile, machine learning is often used to improve the accuracy and prediction of several key genes identified based on traditional microarray or next-generation sequencing data [28]. Recently, the SVM-RFE algorithm and LASSO regression analysis have been the most widely used machine learning methods to identify key genes [29]. Thus, in the current research, WGCNA combined with machine learning methods was used to identify key immune-related genes involved in the pathogenesis of AF. We noticed that the lightcyan and lightgreen modules were significantly associated with infiltrating immune cells, such as regulatory T cells, follicular helper T cells, MDSCs and activated CD8 T cells. Then, a total of 16 overlapping genes were identified by both the SVM-RFE algorithm and LASSO regression analysis. Moreover, we also noticed that the expression levels of *NCF2* and *HCST* were significantly different between control and AF patient samples and among AF patient samples with three distinct m6A modification patterns. Meanwhile, RT-qPCR analysis also proved that the expression of *NCF2* and *HCST* was significantly increased in AF patients compared with SR subjects. These results consistently indicate that *NCF2* and *HCST* are significantly related to the pathogenesis of AF, and elucidating the molecular mechanism of their involvement in AF is expected to provide new molecular targets for the prevention and treatment of AF.

Currently, the *NCF2* gene and its genetic variants have been reported to be associated with susceptibility to a variety of inflammation- or immune-related diseases. Cunninghame et al. found that rs10911363, an intronic variant in the *NCF2* gene, was associated with systemic lupus erythematosus (SLE) susceptibility in a European population [30]. Jiao et al. proved that the rs10911362 variant in the *NCF2* gene was correlated with decreased tuberculosis and pulmonary tuberculosis susceptibility in a Chinese population [31]. In addition, Li et al. suggested that the *NCF2* gene is expected to become a molecular marker for the diagnosis of pulmonary tuberculosis, and its expression was significantly increased in patients with pulmonary tuberculosis [32]. Su et al. found that the *NCF2* gene was significantly overexpressed in advanced atherosclerotic plaques and may play a key role in the development of psoriasis complicated with atherosclerosis [33]. Moreover, some scholars have found that *NCF2* may be a key molecule in patients with nonalcoholic fatty liver disease complicated with AF [34], and the expression of *NCF2* is significantly upregulated in patients with AF [35]. On the other hand, Zhou et al. found that overexpression of immune-related genes, such as *HCST,* was significantly correlated with high infiltration of immune cells, especially dendritic cells, in clear cell renal cell carcinoma (ccRCC), and high expression of *HCST* was significantly correlated with poorer prognosis in ccRCC patients [36]. Wang et al. revealed that *HCST* is significantly associated with some immune cells, such as CD8 T cells, B cells, macrophages, CD4 T cells, dendritic cells and neutrophils, and is involved in several inflammatory or immune-related signaling pathways, including the T-cell receptor signaling pathway, cytokine-cytokine receptor signaling pathway, chemokine signaling pathway, pathways involving cell adhesion molecules, FC gamma-mediated phagocytosis and B-cell receptor signaling pathway, and that overexpression of *HCST* can significantly affect the clinical stage, tumor grade and prognosis of kidney renal clear cell carcinoma (KIRC) [37]. However, the correlation between *NCF2*, *HCST* and immune cells and whether the expression levels of *NCF2* and *HCST* are affected by the m6A modification pattern in AF remain unclear. In the current research, we found that *NCF2* and/or *HCST* were positively correlated with multiple immune cells, excluding CD56dim natural killer cells, and their expression levels were significantly upregulated in the m6A cluster-B and -C groups compared with the m6A cluster-A group. Meanwhile, a positive correlation between *NCF2*, *HCST* and several inflammation- or immune-related HALLMARKS pathways was also observed. These results indicate that *NCF2* and *HCST* are key immune regulatory molecules involved in AF.

The current research had several limitations. First, the validation sample size in this study was small, and the subjects were recruited from a single center, so these findings still need to be validated in other multicenter studies or studies with larger sample sizes. Second, the immune cell analysis in this study adopts the most widely used analysis method to quantify the number of immune cells, but single-cell sequencing is still required to obtain the most accurate number of immune cells. Third, we were unable to obtain more clinical features or serological results of AF samples in the GSE31821, GSE41177, GSE79768 and GSE115574 datasets. Therefore, it is difficult to reveal the key role of m6A modification in immune regulation from multiple perspectives, and the analysis results are relatively singular. Fourth, most of the findings in this study are based on bioinformatics analysis. Although RT-qPCR was used to verify the expression of *NCF2* and *HCST* in clinical samples, the relationship between *NCF2*, *HCST* and immune cells and immune-related pathways still needs to be verified by more *in vitro* and *in vivo* experiments.

In conclusion, m6A modification plays a key role in the complexity and diversity of the immune microenvironment of AF. Immunotyping of patients with AF will help to develop more accurate immunotherapy strategies for patients with a significant immune response. The m6A modification can affect the expression of the immune-related genes *NCF2* and *HCST*, and *NCF2* and *HCST* are expected to become new targets for immunotherapy of AF.

## MATERIALS AND METHODS

### AF microarray datasets

GSE31821, including 6 atrial tissue samples (2 SR and 4 AF samples), GSE41177, including 19 atrial tissue samples (3 SR and 16 AF samples), GSE79768, including 26 atrial tissue samples (12 SR and 14 AF samples), and GSE115574, including 59 atrial tissue samples (31 SR and 28 AF samples), were downloaded from the Gene Expression Omnibus (GEO, http://www.ncbi.nlm.nih.gov/geo). These datasets were based on the GPL570 Affymetrix Human Genome U133 Plus 2.0 array. An integrated gene expression matrix was obtained after normalization and elimination of interbatch differences between GSE31821, GSE41177, GSE79768 and GSE115574. The gene expression profiles of GSE31821, GSE41177, GSE79768 and GSE115574 were normalized by the “*limma*” package [38]. When a probe corresponded to multiple genes at the same time, it was excluded from the analysis. When multiple different probes corresponded to the same gene, we took the average gene expression value detected by those probes as the true expression value of the gene. The interbatch differences between the GSE31821, GSE41177, GSE79768 and GSE115574 datasets were eliminated by the ComBat function in the “sva” package in R software.

### Identification of key m6A regulators

The Wilcoxon test was used to evaluate the expression status differences of 23 m6A regulators between healthy individuals and AF patients. Then, a random forest model was constructed by the “randomForest” package in R and used to identify key m6A regulatory factors. Specifically, the average model error rate of all m6A regulatory factors was calculated, the optimal number of variables of the binary tree in the node was set as 6, and 300 was selected as the optimal number of trees contained in the random forest. Then, the random forest model was constructed, and the decreasing precision method (Gini coefficient method) was used to obtain the dimension importance value from the random forest model. Factors with importance values greater than 2 [39, 40] were selected as key m6A regulators for subsequent model construction.

### Construction and verification of the nomogram

The predictive nomogram was constructed by the “rms” package in R software based on the expression values of six key m6A regulators. Then, the calibration curve was used to assess the predictive power of the nomogram model. Decision curve analysis was used to evaluate the clinical value of the nomogram model. Finally, receiver operating characteristic (ROC) analysis was used to evaluate the diagnostic performance of the nomogram model in distinguishing AF patients from healthy subjects.

### Identification of the m6A modification pattern

Based on the expression of six key m6A regulators, unsupervised clustering analysis was used to identify different m6A modification patterns in AF. The robustness and cluster numbers were calculated by the consensus clustering algorithm [41, 42]. The R package “ConsensuClusterPlus” was utilized to perform the above steps for 1000 iterations to guarantee the robustness of the classification [43]. Principal component analysis (PCA) was used to further verify the different m6A modification patterns distinguished by six key m6A regulators.

### Single-sample gene set enrichment analysis (ssGSEA) and GSVA enrichment analysis

Single-sample gene-set enrichment analysis (ssGSEA) was used to estimate the number of specific infiltrating immune cells between controls and AF patients as well as among distinct m6A modification patterns, which defines an enrichment score to represent the degree of absolute enrichment of a gene set in each sample within a given dataset [44]. The list of infiltrating immunocyte gene sets was obtained from a previous study [41]. The Wilcoxon test was utilized to compare enrichment scores representing immunocyte abundance between different m6A modification patterns.

GSVA enrichment analysis was used to evaluate the 50 HALLMARK pathways between control and AF patient samples as well as among AF patient samples with three distinct m6A modification patterns via the ‘GSVA’ package in R software. The gene sets of ‘h.all.v7.0.symbols’ were extracted from the MSigDB database [45] (http://software.broadinstitute.org/gsea/msigdb/index.jsp) to run GSVA. Then, the correlations between hub genes and infiltrating immune cells and 50 HALLMARK pathways were determined by Spearman correlation analysis.

### WGCNA analysis

A scale-free network based on a gene expression matrix was constructed using WGCNA, which is one of the most commonly used tools in systems biology [46]. Genes with the top 25% of variance were selected for the WGCNA. In the current research, the appropriate soft threshold was defined as 14, and detailed WGCNA steps refer to the method described in our recently published article [47].

### Enrichment analysis of genes in meaningful modules

Kyoto Encyclopedia of Genes and Genomes (KEGG) and gene ontology (GO) functional enrichment analyses based on the expression profile of genes in the meaningful modules were performed by clusterProfer and the DOSE package [48]. The threshold was defined as FDR < 0.05.

### Identification of key genes using machine learning

At present, the least absolute shrinkage and selector operation (LASSO) and support vector machine-recursive feature elimination (SVM-RFE) algorithms are the two most commonly used machine learning methods for identifying key genes with the best prognostic value for disease. Based on the expression profile of genes in the meaningful modules, the “glmnet” package in R was used to perform LASSO logistic regression analysis [49]. In addition, SVM-RFE acts as an effective feature selection technique that finds the best variables by deleting the feature vector generated by SVM [50]. The selected biomarkers in the diagnosis of AF were analyzed and classified by the SVM classifier based on the SVM function in the e1071 package. The overlapping key genes identified by the above machine learning methods were defined as hub genes. The differential expression of hub genes between controls and AF patients as well as among AF samples with several distinct m6A modification patterns was analyzed by t test and analysis of variance (ANOVA), respectively.

### Study population

A total of 91 SR subjects and 86 persistent AF patients were recruited from the Cardiology Department of Hunan Provincial People’s Hospital. AF lasting more than 7 days was defined as persistent AF [51]. Patients with a history of type 1 diabetes, hematologic disease, hypertension, coronary heart disease, neoplasia, autoimmune disease, and renal or liver diseases were excluded.

### RT-qPCR

According to the manufacturer’s instructions, total RNA was extracted from peripheral blood using the UNlQ-10 Column TRIzol Total RNA Isolation Kit (Sangon Biotech, Shanghai, China). The purity and concentration of the extracted RNA was checked by a NanoDrop 2000 Spectrophotometer (Thermo Fisher Scientific, Waltham, MA, USA), with an A260/A280 between 1.8 and 2.0. The cDNA was reverse-transcribed using the PrimeScriptTM RT Reagent Kit (Takara, Otsu, Japan). Using GAPDH as a reference, we performed quantitative RT-PCR on an ABI 7500 instrument (Applied Biosystems, Waltham, MA, USA) using a Taq PCR Master Mix Kit (Takara, Otsu, Japan). Primer sequences for hub genes and references were designed and validated by Songon Biotech (Songon Biotech, Shanghai, China). The 2^−ΔΔCt^ method was used to calculate the relative expression level of hub genes. The t test was used to compare the expression of hub genes between controls and AF patients. The ROC curves were constructed based on the expression levels of the hub genes.

## Supplementary Material

Supplementary Figures

Supplementary Table 1

Supplementary Table 2

Supplementary Table 3

Supplementary Table 4

Supplementary Table 5

Supplementary Table 6

Supplementary Table 7

Supplementary Table 8
