# Supplementary material for: Identification of m6A regulator-mediated RNA methylation modification patterns and key immune-related genes involved in atrial fibrillation
Source: Aging (Albany NY). 2023 Feb 20;15(5):1371–93. doi: 10.18632/aging.204537 (PMC10042702; doi:10.18632/aging.204537)
Supplement: Supplementary Figures [file aging-15-204537-s001.pdf]

SUPPLEMENTARY FIGURES

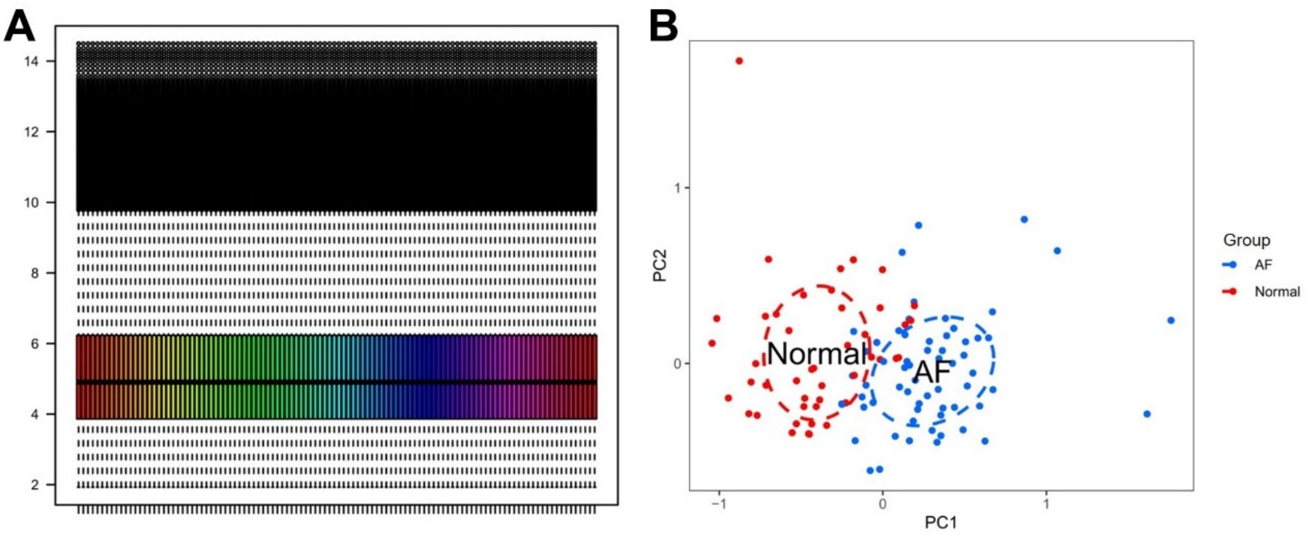

Supplementary Figure 1. Standardization and principal component analysis of all samples. (**A**) Normalized for all samples. (**B**) Principal component analysis for all samples.

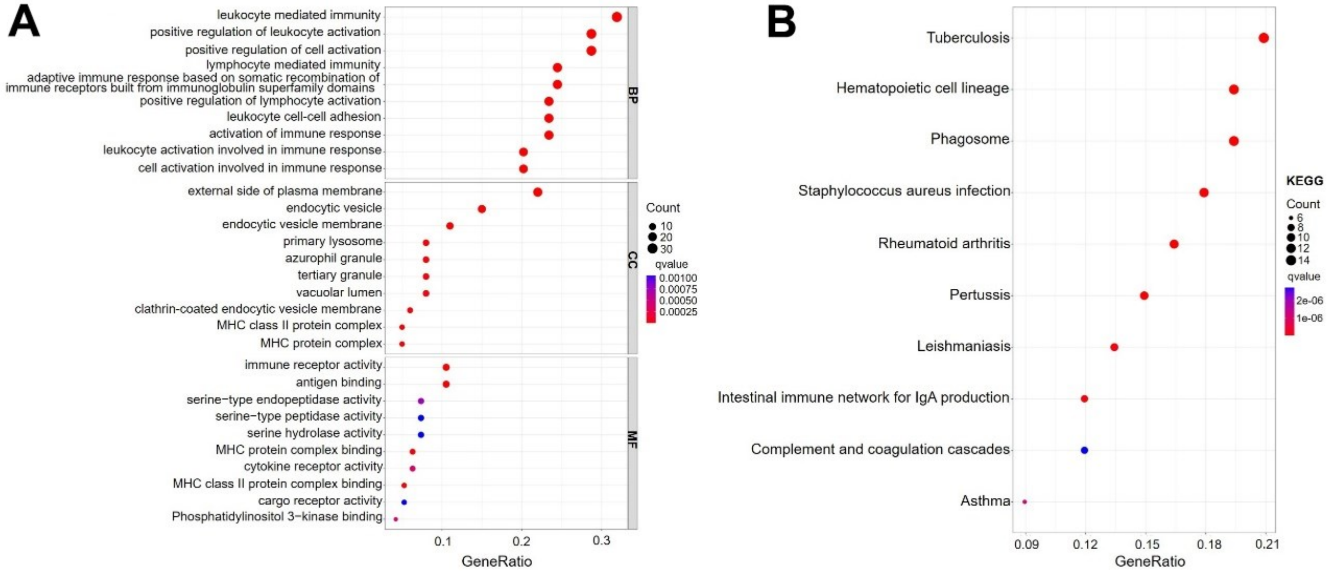

Supplementary Figure 2. Enrichment analysis of the genes in the meaningful modules. (**A**) GO functional enrichment analysis. (**B**) KEGG pathways analysis. The gene number was represented at x-axis, the KEGG pathway and GO terms were presented at y-axis.

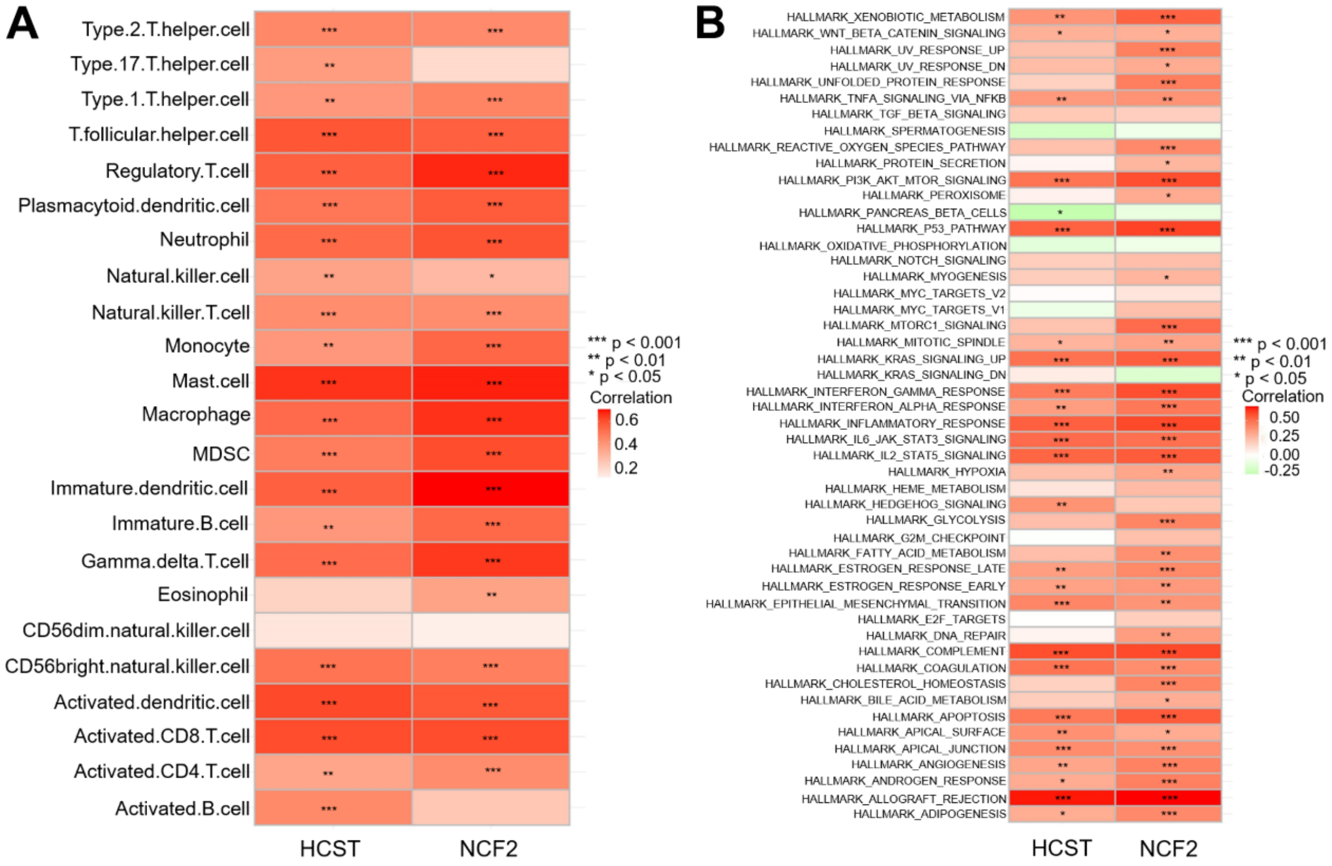

Supplementary Figure 3. Heatmap of correlations between *NCF2* as well as *HCST* genes and infiltrating immune cells and 50 HALLMARKS pathways. (**A**) The relationship between infiltrating immune cells and the *NCF2* and *HCST* genes. (**B**) The relationship between 50 HALLMARKS pathways and the *NCF2* and *HCST* genes. *$P < 0.05$; **$P < 0.01$; ***$P < 0.001$.
