# Supplementary material for: Identification of m6A regulator-mediated RNA methylation modification patterns and key immune-related genes involved in atrial fibrillation
Source: Aging (Albany NY). 2023 Feb 20;15(5):1371–93. doi: 10.18632/aging.204537 (PMC10042702; doi:10.18632/aging.204537)
Supplement: Supplementary Table 2 [file aging-15-204537-s003.pdf]

Supplementary Table 2. The m6A RNA methylation modification patterns mediated by 6 key m6A regulators in AF.

ID	RBM15B	HNRNPC	HNRNPA2B1	IGFBP2	IGFBP3	ALKBH5	m6Acluster
GSM789271_treat	5.90138	7.129	8.058032	9.083843	10.027447	7.460989	A
GSM789272_treat	6.585327	7.377221	7.066014	10.813303	10.0168	7.8383	B
GSM789274_treat	6.258503	7.531016	8.161363	9.405052	10.37481	7.624308	C
GSM789275_treat	6.474373	7.389506	7.228629	9.302356	9.066857	7.742908	C
GSM1005424_treat	6.262892	7.408608	7.810999	10.117958	9.868154	7.598898	C
GSM1005426_treat	6.412639	7.339644	7.8383	10.299514	9.623874	7.605746	C
GSM1005428_treat	6.435906	7.372142	7.328194	10.48625	9.491856	7.665631	B
GSM1005430_treat	6.430108	7.380779	7.778062	9.810857	9.88699	7.572645	C
GSM1005432_treat	6.261587	7.384813	7.804135	9.435905	9.538878	7.474808	C
GSM1005434_treat	6.343933	7.352928	7.663704	9.979509	9.990233	7.55616	C
GSM1005436_treat	6.409742	7.402653	7.443494	9.88699	10.092063	7.580118	C
GSM1005438_treat	6.291567	7.39058	7.567494	9.86198	10.066885	7.515343	C
GSM1005440_treat	6.359973	7.374409	7.30202	9.881155	9.963446	7.605746	C
GSM1005442_treat	6.270799	7.399298	7.722304	9.841396	9.57037	7.532237	C
GSM1005444_treat	6.490407	7.477738	7.340784	10.257411	9.510538	7.75741	C
GSM1006245_treat	6.354654	7.442932	7.726283	9.706861	10.114567	7.634785	C
GSM1006247_treat	6.632033	7.283248	7.904238	10.287115	9.906506	7.543983	C
GSM1006249_treat	6.542227	7.186585	7.930539	10.037417	9.349632	7.230754	C
GSM1006251_treat	6.185407	7.41665	7.782726	9.792959	9.588153	7.390041	C
GSM1006253_treat	6.26602	7.377221	7.648592	9.792959	9.445125	7.49398	C
GSM2102184_treat	6.153389	7.511045	7.989245	10.918397	10.166073	7.630532	B
GSM2102185_treat	6.206053	7.49464	7.663039	10.519623	10.941273	8.067351	B
GSM2102186_treat	6.285993	7.525887	7.67074	9.477383	8.425224	7.621636	C
GSM2102187_treat	6.091918	7.607638	8.185945	8.479291	9.242633	7.563582	A
GSM2102188_treat	6.127192	7.277583	7.887248	9.38702	9.69243	6.942886	C
GSM2102189_treat	6.225165	7.273687	7.982342	9.661232	10.536379	7.736113	C
GSM2102190_treat	6.605199	7.331015	7.529836	10.429378	8.932519	7.53881	B
GSM2102191_treat	6.558832	6.843365	7.640226	9.289926	9.755126	7.711509	C
GSM2102192_treat	6.473328	7.131818	7.560531	9.760976	9.11236	7.349055	C
GSM2102193_treat	6.206053	7.380779	7.962586	8.014668	10.450505	7.786423	A
GSM2102194_treat	6.541586	7.331607	7.565579	9.912989	8.996157	7.69189	C
GSM2102195_treat	6.577509	7.306838	6.94679	9.859129	9.332328	8.33254	C
GSM2102196_treat	6.266912	7.335861	7.658575	6.649301	9.947684	7.859217	A
GSM2102197_treat	6.928074	6.971467	7.782726	9.183712	8.754232	7.649273	C
GSM3182680_treat	6.451409	7.344628	7.666274	10.502601	8.380627	7.747689	B
GSM3182681_treat	6.29024	7.246575	7.339644	9.629004	9.993466	7.839076	C
GSM3182682_treat	6.116225	7.191712	7.366893	10.723776	8.908684	7.810999	B
GSM3182683_treat	6.331233	7.402653	7.520917	10.051576	9.925384	7.595759	C
GSM3182684_treat	6.565227	7.342487	7.717524	10.799913	9.144803	7.608857	B
GSM3182685_treat	6.123798	7.309552	7.675719	8.650796	10.084928	7.829262	A
GSM3182686_treat	6.441962	7.041962	7.582069	10.910656	9.046697	7.399848	B
GSM3182687_treat	6.473962	7.263157	7.521482	9.51298	9.906506	7.780009	C
GSM3182688_treat	6.34714	7.189202	7.48677	11.323854	10.102326	7.601435	B
GSM3182689_treat	6.515523	7.527979	8.163929	10.949486	9.631794	7.125972	B
GSM3182690_treat	6.126871	7.380779	7.749103	10.799913	9.943886	7.272573	B
GSM3182691_treat	6.384972	7.450711	7.957127	9.732656	10.491708	7.6409	C
GSM3182692_treat	6.624983	7.20753	8.019662	8.689859	9.43333	7.210897	A
GSM3182693_treat	6.408093	7.499333	7.663039	9.051927	10.261423	7.252255	A

GSM3182694_treat	5.759048	7.147268	7.850979	11.034949	8.870444	6.644807	B
GSM3182695_treat	6.218431	6.932924	7.709471	10.577904	9.996985	7.522144	B
GSM3182696_treat	6.896041	6.749387	6.905003	11.061198	9.426002	7.398668	B
GSM3182697_treat	6.243748	6.926863	7.101934	11.968874	11.093496	7.864051	B
GSM3182698_treat	6.203795	7.425675	7.812532	11.755773	9.300529	8.211354	B
GSM3182699_treat	5.949588	7.403739	7.410937	11.981442	11.248291	8.009548	B
GSM3182700_treat	5.91652	7.239622	8.155872	9.897344	8.386245	7.374409	C
GSM3182701_treat	6.04131	7.221645	7.688407	8.574566	10.730452	7.976127	A
GSM3182702_treat	6.290576	7.355592	7.309552	10.641517	9.20503	7.940356	B
GSM3182703_treat	6.837514	7.388342	7.407493	10.903509	10.460398	8.209532	B
GSM3182704_treat	6.74385	7.222691	7.660359	11.350736	9.260431	7.323158	B
GSM3182705_treat	6.525857	7.165862	8.441135	6.14668	10.778199	5.754425	A
GSM3182706_treat	6.083648	6.797624	7.952319	8.57177	10.218241	7.377736	A
GSM3182707_treat	6.333223	6.878329	7.695903	9.684138	7.761619	7.504573	B
