# Supplementary material for: Identification of m6A regulator-mediated RNA methylation modification patterns and key immune-related genes involved in atrial fibrillation
Source: Aging (Albany NY). 2023 Feb 20;15(5):1371–93. doi: 10.18632/aging.204537 (PMC10042702; doi:10.18632/aging.204537)
Supplement: Supplementary Table 8 [file aging-15-204537-s009.pdf]

Supplementary Table 8. The details genes symbols of several key genes identified by two distinct machine learning methods.

Common elements in LASSO and SVM-RFE :
AMICA1
BCAT1
C16orf54
C1QC
CD14
CD48
CD8A
CORO1A
CXCL12
HCLS1
HCST
HMHA1
IGLV1-44
LAPTM5
NCF2
TNFSF13
Elements only in LASSO :
C1S
CASP1
CD2
CTSS
GZMH
HLA-DMB
HPGDS
ITGAM
LCK
LYZ
MS4A7
SLC7A7
VAMP8
Elements only in SVM-RFE :
HLA-DRA
LYN
HLA-DMA
LYVE1
IGLC1
CCL5
CD163
GIMAP2
RNASE6
MARCKS
CCL19
MRC1
